# Prevalence of chronic traumatic encephalopathy in the Sydney Brain Bank

**DOI:** 10.1093/braincomms/fcac189

**Published:** 2022-08-01

**Authors:** Heather McCann, Anita Y Bahar, Karim Burkhardt, Andrew J Gardner, Glenda M Halliday, Grant L Iverson, Claire E Shepherd

**Affiliations:** Neuroscience Research Australia, Randwick, NSW 2031, Australia; Neuroscience Research Australia, Randwick, NSW 2031, Australia; School of Medical Sciences, University of New South Wales, Kensington, NSW 2052, Australia; School of Medicine and Public Health, College of Health, Medicine and Wellbeing, The University of Newcastle, Callaghan, NSW 2308, Australia; Neuroscience Research Australia, Randwick, NSW 2031, Australia; Faculty of Medicine and Health School of Medical Sciences, University of Sydney Brain and Mind Centre, Camperdown, NSW 2050, Australia; Department of Physical Medicine and Rehabilitation, Harvard Medical School, Boston, MA 02114, USA; Department of Physical Medicine and Rehabilitation, Spaulding Rehabilitation Hospital, Charlestown, MA 02114, USA; Home Base, A Red Sox Foundation and Massachusetts General Hospital Program, Charlestown, MA 02114, USA; MassGeneral Hospital for Children Sports Concussion Program, Boston, MA 02114, USA; Neuroscience Research Australia, Randwick, NSW 2031, Australia; School of Medical Sciences, University of New South Wales, Kensington, NSW 2052, Australia

**Keywords:** chronic traumatic encephalopathy, phosphorylated tau, traumatic brain injury

## Abstract

Chronic traumatic encephalopathy neuropathologic change can only be definitively diagnosed post-mortem. It has been associated with repetitive mild neurotrauma sustained in amateur and professional contact, collision and combat sports, although it has also been documented in people with a single severe traumatic brain injury and in some people with no known history of brain injury. The characteristic neuropathology is an accumulation of perivascular neuronal and astrocytic phosphorylated tau in the depths of the cortical sulci. The tau-immunopositive neurons and astrocytes that are considered pathognomonic for chronic traumatic encephalopathy are morphologically indistinguishable from Alzheimer-related neurofibrillary tangles and ageing-related tau astrogliopathy, respectively, although they are found in different spatial distributions throughout the cortex. The Sydney Brain Bank collection consists of neurodegenerative diseases and neurologically normal controls. We screened 636 of these cases for chronic traumatic encephalopathy neuropathologic change. A subset of 109 cases had a known history of traumatic brain injury. Three cortical regions were screened for the presence of neuronal and astrocytic phosphorylated tau according to the current 2021 National Institute on Neurological Disorders and Stroke/National Institute of Biomedical Imaging and Bioengineering consensus criteria for chronic traumatic encephalopathy. Five cases (0.79%) showed pathological evidence of chronic traumatic encephalopathy and three of these had a history of traumatic brain injury. Three cases had coexisting Alzheimer’s and/or Lewy body disease pathology meeting criteria for neurodegenerative disease. Another eight cases almost met criteria for chronic traumatic encephalopathy neuropathological change except for an absence of neuronal tau or a strict perivascular arrangement. Ageing-related tau astrogliopathy was found in all eight cases as a coexisting neuropathology. Traumatic brain injury was associated with increased odds ratio [1.79, confidence interval 1.18–2.72] of having a higher neurofibrillary tangle stage and phosphorylated TAR DNA binding protein 43 (OR 2.48, confidence interval 1.35–4.54). Our study shows a very low rate of chronic traumatic encephalopathy neuropathological change in brains with or without neurodegenerative disease from the Sydney Brain Bank. Our evidence suggests that isolated traumatic brain injury in the general population is unlikely to cause chronic traumatic encephalopathy neuropathologic change but may be associated with increased brain ageing.

## Teaser

Recent studies suggest that chronic traumatic encephalopathy (CTE) neuropathologic change might be very uncommon in the general population. McCann *et al*. report a very low rate (<1%) of CTE neuropathologic change in the brains of 636 individuals with or without neurodegenerative disease from the Sydney Brain Bank.

## Graphical Abstract

**Graphical Abstract:**
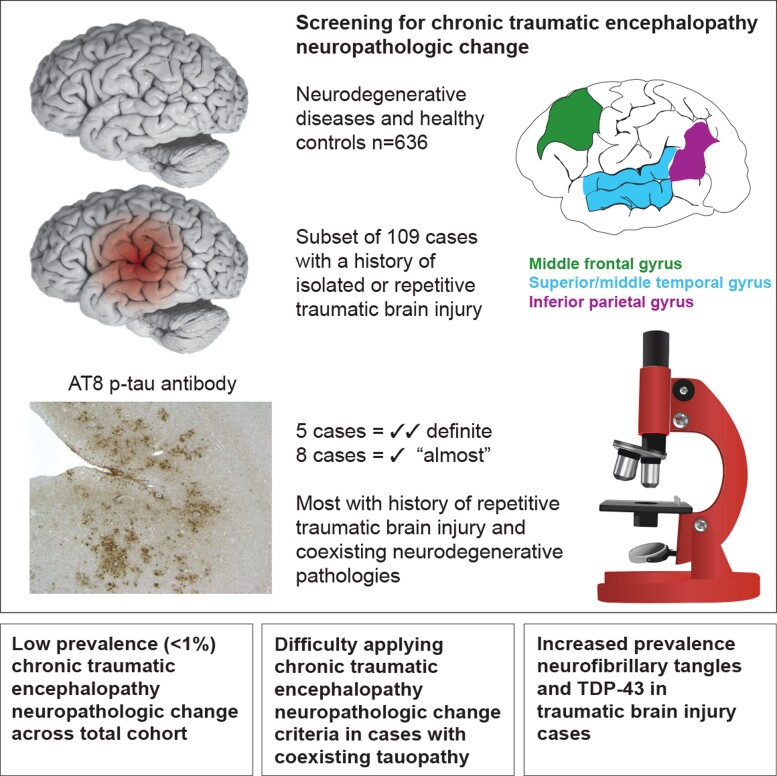

## Introduction

The relationship between repetitive traumatic brain injury (TBI) and a neurological condition was first recognized in the early 1900s with the description of ‘punch drunk’ syndrome,^1,2^ traumatic encephalopathy^3^ and dementia pugilistica^3,4^ in boxers. Omalu *et al*.^5,6^ initially reported post-mortem neuropathology in case studies of professional American football players and a professional wrestler. Other studies have reported chronic traumatic encephalopathy neuropathologic change (CTE-NC) in individuals who participate in Australian rules football, Australian rugby league, rugby union, soccer, baseball, ice hockey and in cases of military personnel subjected to blasts.^7–14^ In 2014, new research criteria for diagnosing traumatic encephalopathy syndrome, the clinical condition hypothesized to be associated with CTE-NC, were published.^15^ Those criteria for traumatic encephalopathy syndrome were replaced by new criteria in 2021, developed through an interdisciplinary consensus process and those new criteria provide a provisional level of certainty for CTE-NC.^16^

Since 2011, several studies have proposed guidelines for the identification of the pathognomonic lesions of CTE-NC.^17–19^ In 2016, a consensus panel of neuropathologists agreed that neuronal and astrocytic phosphorylated tau pathology located in a perivascular distribution in the sulcal depths was indicative of CTE-NC.^18^ Other supportive features proposed from both this study and a subsequent consensus study in 2021^19^ are the presence of neuronal tau in superficial cortical layers and the CA2 and CA4 regions of the hippocampus, subcortical neuronal and astrocytic tau and tau grains. Non-tau pathological supportive features include TAR DNA binding protein 43 (TDP-43) positive inclusions and neurites in the medial temporal lobe, macroscopic changes consisting of cortical and subcortical brain atrophy, enlargement of the 3rd ventricle, cavum septum pellucidum and mammillary body atrophy.^10,18,19^ Four progressive stages of pathological severity were described in 2013,^10^ but these were not validated in the 2016 consensus study^18^ or the most recent 2021 consensus study^19^ and as such they are not a feature of the current diagnostic guidelines. Instead, a pathology severity grade has been proposed that characterizes a case as ‘low’ or ‘high’ CTE-NC based on the distribution of neuronal tau throughout cortical and subcortical regions.^19^

It is not clear how much CTE-NC is required to manifest clinical disease, and both cardinal and supportive pathological features of CTE-NC (neuronal tau and TDP-43-positive inclusions and neurites) are widely seen in other neurodegenerative conditions and the normal aged brain. Multiple studies have shown that not all cases with a history of repetitive mild neurotrauma in sports display CTE-NC. Using the first consensus diagnostic recommendations,^18^ CTE-NC was reported in 66–87% of former professional American football players,^20^ Scottish soccer and Rugby players^21^ and English professional soccer players.^22^ Other studies of former professional athletes have shown lower percentages of CTE-NC (48–50%) in professional Canadian football and ice hockey players.^23,24^ However, all of the above studies of former athletes are not population-based, with potential bias in those coming to post-mortem brain examination. Indeed, studies analysing brain bank cohorts have reported much lower levels of CTE-NC (12–31.8%) in individuals with a history of participation in contact sports.^25,26^ CTE-NC has also been found in a routine neuropathology service in Canada in individuals with a history of head injury with or without substance abuse^27^ and a brain bank population in the UK in individuals without a known history of head injury and no neurological symptoms.^28^

There have been few large-scale investigations of CTE-NC and most studies in neurodegenerative brain bank cohorts were performed before the development of the first consensus criteria.^18^ These studies have reported varying percentages of CTE-NC. A large-scale study of 450 decedents who did not participate in youth sports found 3% of cases had CTE-NC,^25^ while another found a higher rate of 11.9% in 268 cases.^28^ In a large community-based sample of 532 cases, only three cases (0.6%) had CTE-NC and none of these cases had a known history of head injury with loss of consciousness or participation in contact sports.^29^ Another community-based study found no evidence of CTE-NC, using a strict definition of CTE-NC, in their cases.^30^

We have assessed cortical regions of 636 neurodegenerative disease and normal healthy control cases in the Sydney Brain Bank to ascertain the prevalence of CTE-NC using both the first and second consensus criteria.^18,19^ A subset of cases was identified with a history of TBI with or without loss of consciousness and a small proportion of these cases also had a history of contact sport played regularly at the professional or club level. We provide evidence of low prevalence of CTE-NC, even in cases with history of TBI.

## Materials and methods

### Case recruitment

Cases were recruited through prospective brain donor programmes with a focus on ageing and neurodegeneration. As such, they are representative of patients with neurodegenerative conditions and healthy controls in our local region of NSW, Australia. The Sydney Brain Bank has ethical approval from the University of New South Wales, Australia, to collect, characterize, store and distribute human brain tissue for the purpose of medical research. The broad case types can be seen in Table 1. All cases were screened based on current published neuropathological diagnostic criteria^31–44^ and using a standardized panel of histologically and immunohistochemically stained neuroanatomical regions. The routine screening comprises sections sampled from (i) superior frontal, pre-central, inferior temporal and anterior cingulate gyri; (ii) hippocampus, entorhinal gyrus and amygdala; (iii) midbrain, pons and medulla and (iv) caudate, putamen and cerebellar dentate. These regions were stained with haematoxylin and eosin, modified Bielschowsky silver and antibodies to detect phospho-tau, ß-amyloid, p62, α-synuclein and TDP-43 proteins. Additional sampling and staining were performed where indicated by clinical presentation or gross observations of abnormalities during macroscopic examination. Where a case had evidence of more than one neuropathology meeting criteria for neurodegenerative disease, the dominant neuropathology was listed as the primary diagnosis (Table 1). More than one type of pathology, and diagnosis, was common.

**Table 1:** Primary neuropathological diagnosis (*n* = 636) and presence of CTE-NC

Case type	Sample size	% Males	Mean age	CTE-NC (%)
Alzheimer’s disease neuropathologic change—intermediate and high	176	52.3	77 ± 14	1.1
Lewy body disease (dominant movement disorder)	111	68.5	80 ± 7	0
Lewy body disease (dominant dementia)	35	74.3	80 ± 9	2.9
Multiple system atrophy	22	54.5	70 ± 8	0
Frontotemporal lobar degeneration with TDP-43	52	51.9	69 ± 9	0
Motor neuron disease	55	63.6	64 ± 12	0
Progressive supranuclear palsy	55	58.2	77 ± 7	0
Corticobasal degeneration	16	62.5	71 ± 11	0
Pick’s disease	8	62.5	74 ± 5	0
Globular glial tauopathy	12	58.3	73 ± 11	0
Rare diseases, including frontotemporal dementia with FUS, neuronal intranuclear inclusion disease	27	59.3	81 ± 16	0
Huntington’s disease	15	73.3	62 ± 17	0
Cerebrovascular disease	29	55.2	88 ± 10	0
Healthy aged controls	21	42.9	86 ± 9	0
Chronic traumatic encephalopathy neuropathologic change	2	100	79 ± 2	100

Two cases had already been identified as having CTE-NC as the primary diagnosis during our routine neuropathological screen. A total of 636 cases were screened. The majority of cases were sporadic in origin (*n* = 507), although some had either a known genetic mutation or a familial history consistent with a dominantly inherited disorder (*n* = 129, see Table 1 for case types, numbers, gender and age distributions). Clinical diagnosis of neurodegenerative disease and injury were collected prospectively from recruitment via clinical notes or questionnaires completed by the participant, their next-of-kin or their clinician. Any history of TBI was collected retrospectively at the time of recruitment via specific questions, including whether there had ever been a head injury, the year the head injury was sustained and further details of head injury. Additional information regarding sporting career or military service was obtained retrospectively from publicly available obituaries. As a rule, we did not know whether, or the extent to which, cases participated in contact, collision or combat sports during adolescence or early adulthood; therefore, these cases are underestimated. However, a small number of people had a documented history of participation in combat (e.g. boxing) or collision (e.g. rugby) sports. These individuals had participated in either organized senior club level or professional sport. In total, 109 cases were identified as having a previous TBI (17.1% of the cohort) and of these 17 (15.6%) had documented TBI-related loss of consciousness. Seven of the 109 TBI cases (6.4%) also had a documented history of contact sport participation. We did not systematically collect (or have access to) information relating to the severity of TBI, whether it was associated with structural findings on neuroimaging, the functional deficits associated with the injury or the extent to which the person recovered, clinically, from the injury. Information on number of TBIs and duration of loss of consciousness was also not systematically collected, therefore, cases were broadly dichotomized as having a history of TBI or not.

### Tissue sampling and immunohistochemistry for analysis of CTE-NC

In order to detect the pathognomonic cortical lesions according to the 2016 and 2021 National Institute on Neurological Disorders and Stroke/National Institute of Biomedical Imaging and Bioengineering (NINDS/NIBIB) consensus criteria,^18,19^ tissue blocks were retrospectively sampled from the middle frontal gyrus, superior and middle temporal gyrus and inferior parietal gyrus and included as many sulcal depths as possible. A subset of 90 cases were sampled bilaterally, where both formalin-fixed brain hemispheres were available, to ascertain whether there was significant pathological asymmetry. These included cases representative of the neurodegenerative cohort, including sporadic and familial intermediate and high Alzheimer’s disease neuropathologic change, sporadic and familial Lewy body disease, cerebrovascular disease and genetic frontotemporal lobar degeneration with TDP-43. Paraffin-embedded sections were cut on a rotary microtome at 10 µm thickness and stained with a primary antibody to detect phospho-PHF tau (AT8, 1:1500, cat #MN1020, Thermo Scientific, Rockford, IL, USA) using a BenchMark GX autostainer and an Optiview Detection kit (cat# 760-700 Roche Diagnostics).

Cases that were identified as having CTE-NC then had a full set of regions stained according to the 2016 and 2021 NINDS/NIBIB consensus criteria.^18,19^ Where possible, bilateral regions were examined from the cortex, hippocampus, amygdala, basal ganglia, thalamus, midbrain with substantia nigra, pons with locus coeruleus, medulla oblongata, cerebellar cortex, cerebellar dentate nucleus, hypothalamus and mammillary body. Pathology staging was performed based on both 2013 criteria (staging from one to four)^10^ and the 2021 (low or high)^19^ NINDS/NIBIB consensus criteria.

### Microscopic analysis

Using a Zeiss Axioscope A1 brightfield microscope, three investigators (one clinical neuropathologist and two research neuropathologists with over 50 years of collective experience in neurodegenerative pathologies) assessed the presence of tau-immunopositive neurons and astrocytes and noted their distribution within each tissue section, specifically whether they were situated in the sulcal depth in a perivascular arrangement—described as ‘around small vessels’ in the current NINDS/NIBIB consensus diagnostic criteria.^19^ According to the current 2021 consensus criteria, the minimum threshold for the neuropathological diagnosis of CTE-NC is the presence of a single pathognomonic lesion in the cortex, and that lesion consists of phosphorylated tau aggregates in neurons, with or without glial tau in thorn-shaped astrocytes, at the depth of a cortical sulcus around a small blood vessel. The presence of thorn-shaped or granular/fuzzy astrocytes indicating ageing-related tau astrogliopathy (ARTAG)^40^ was assessed in these regions morphologically (not through co-labelling with an astrocytic marker) and the distribution noted, specifically whether it was situated in subpial, subependymal, white matter or cortical regions and whether the arrangement was perivascular. Investigators showed 93.3% agreement when identifying CTE-NC, pathology almost meeting criteria for CTE-NC and ARTAG (Cohen’s kappa = 0.865, indicating almost perfect agreement).

### Statistical analysis

A multivariate ordinal regression model with multivariate logit link, as implemented in the mvord R package,^45^ was used to assess the effect of TBI, age (mean centred average), gender and post-mortem delay on pathological features of common neurodegenerative diseases: amyloid plaques (A Score 0–3), neurofibrillary tangles (B Score 0–3), Lewy pathology (LB Stage 0–6), the presence of cortical ARTAG (yes/no) and limbic-predominant age-related TDP-43 encephalopathy (LATE) (yes/no). The presence or absence of CTE-NC (either as a primary, *n* = 2 or secondary diagnosis, *n* = 3) was not included in the analysis because the number of cases was too low.

### Data availability

The data that support the findings of this study are available from the corresponding author upon reasonable request.

## Results

### Prevalence of CTE-NC

Of the 636 cases screened, five showed tau-immunopositive neuronal and astrocytic pathology located in a strictly perivascular arrangement in the sulcal depths, definitively meeting current criteria for CTE-NC.^19^ This included two cases that had already been identified during routine screening, giving an overall frequency of 0.79% of cases with CTE-NC in our brain bank cohort (Table 1). Of the five cases identified, two had a primary diagnosis of Alzheimer’s disease neuropathological change and one case had limbic Lewy body disease (Table 2). All cases had TDP-43 pathology in the medial temporal lobe consistent with LATE. The two cases with a primary diagnosis of CTE-NC also had hippocampal sclerosis and one case had a small subcortical infarct. Varying severity of ß-amyloid pathology was found in all cases, including those where the primary diagnosis was CTE-NC (Cases 3 and 4, Table 2). No cases meeting strict neuropathological criteria for CTE-NC were found in any other disease group (Table 1).

**Table 2:** Details of cases with CTE-NC

Case number	Age at death	Gender	Disease duration (years)	A score (β-amyloid plaques)	B score (neurofibrillary tangles)	C score (neuritic plaques)	Lewy body stage	TDP-43 pathology	CTE staging 2013	CTE staging 2021	Neuropathological diagnosis
Case 1	89	Female	4	A3	B3	C2	V	LATE stage 2	3	Could not be reliably staged due to the presence of coexisting pathologies	High AD-NC Limbic LBD LATE CTE-NC
Case 2	74	Male	8	A2	B1	C1	VI	In entorhinal cortex only—does not fit staging criteria	1	High (6)	Neocortical LBD LATE CTE-NC
Case 3	78	Male	15	A1	B3	C1	0	LATE Stage 3	4	High (8)	CTE-NC Striatal infarct Hippocampal sclerosis LATE
Case 4	81	Male	2	A1	B3	C0	0	LATE Stage 3	3	High (10)	CTE-NC Hippocampal sclerosis LATE
Case 5	92	Male	2	A3	B2	C2	0	LATE Stage 2	4	Could not be reliably staged due to presence of coexisting pathologies	Intermediate AD-NC LATE CTE-NC

AD-NC, Alzheimer’s disease neuropathologic change; A score, β-amyloid plaque distribution, A0–A3; B score, neurofibrillary tangle distribution, B0–B3; C score, CERAD score, C0–C3; CTE-NC, chronic traumatic encephalopathy neuropathologic change; LATE, limbic-predominant age-related TDP-43 encephalopathy; LBD, Lewy body disease; TBI, traumatic brain injury.

The staging of CTE-NC varied from Stage 1 to 4 using the 2013 staging criteria^10^ (Table 2). Only Cases 2, 3 and 4 could be reliably staged according to the 2021 consensus criteria,^19^ as they did not have a coexisting tauopathy (Table 2). While we attempted to apply the 2021 staging to the remaining two cases, they were all identified as having high CTE-NC due to the presence of neuronal tau throughout all cortical layers, all sectors of the hippocampal formation, and subcortically in the amygdala, mammillary bodies and thalamus. Thus, we were unable to distinguish the neuronal tau restricted to the upper cortical layers and CA2/CA4 sectors of the hippocampus required to stage according to the 2021 consensus criteria.^19^

**Figure 1:**
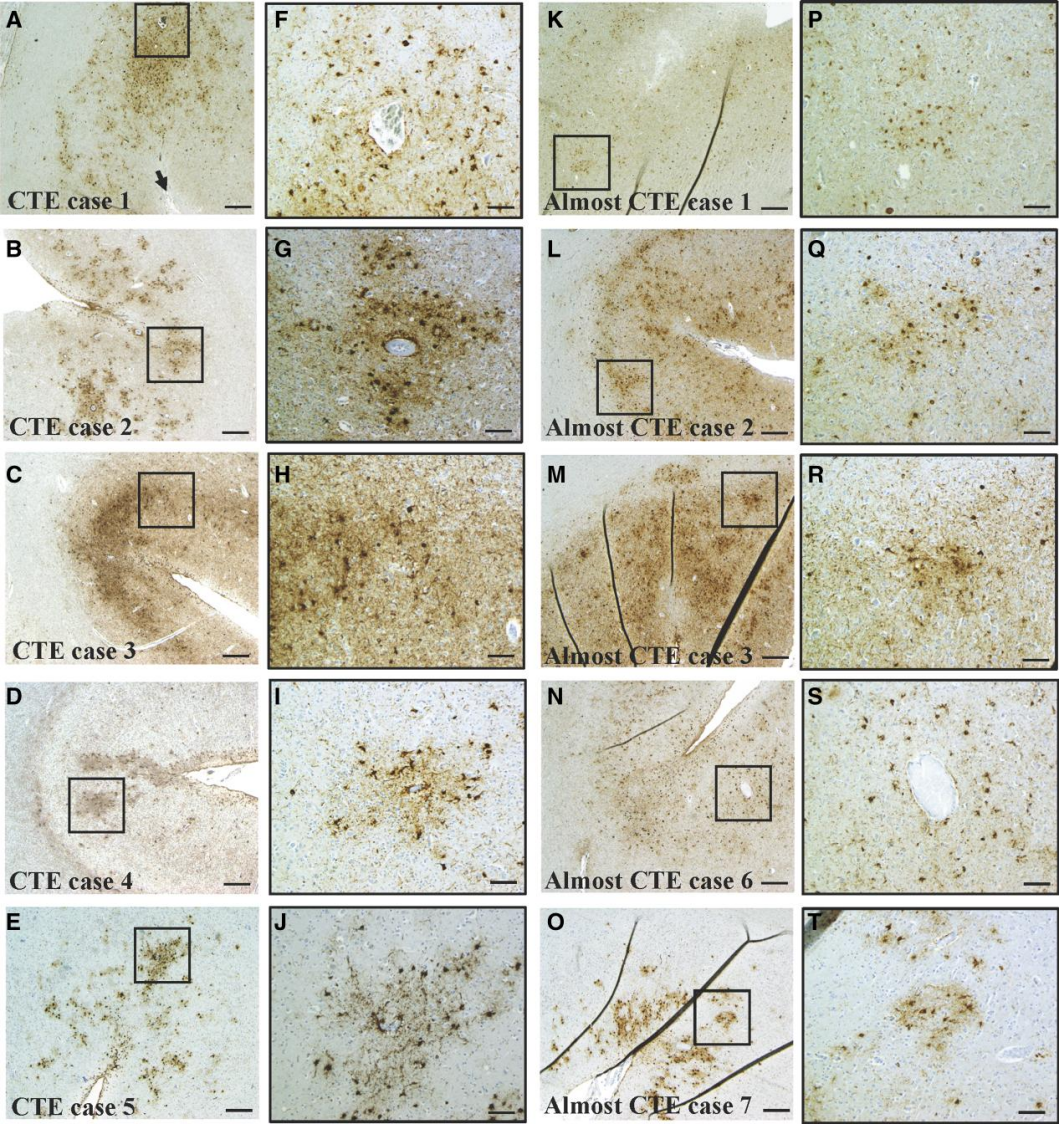
**Representative images of definite and almost CTE-NC.** (**A**–**E**) CTE-NC in cases with definite tau-immunopositive neuronal and astrocytic perivascular lesions at the depth of the sulcus. Black boxes indicate the location of the high magnification image on the right (**F**–**J**). Black arrow on **A** indicates pial surface of sulcus. (**K**–**O**) Representative images of five of the eight cases that almost met criteria for CTE-NC, except for a lack of either a perivascular distribution (**K**, **L**, **M**, **O**) and/or lack of a tau-immunopositive neuronal component (**K**, **N**, **O**). Black boxes indicate the location of the high magnification image on the right (**P**–**T**). **B**, **C**, **D**, **K**, **M**, **N**, **O**, middle frontal gyrus; **A**, superior/middle temporal gyrus; **E** and **L**, inferior parietal gyrus. Scale bars **A**–**E** = 200 µm; **F**–**J** = 50 µm.

The severity of CTE-NC ranged from one focal lesion (Case 2), to isolated focal lesions in two separate sampled regions (Case 1), to multiple foci in all three sampled cortical regions (Cases 3–5). CTE-NC was found in the middle frontal and superior/middle temporal gyri in four of the five cases, with lesions in the inferior parietal gyrus found in three cases. Of the two cases with bilateral samples, one had bilateral lesions in the middle frontal gyrus and left-sided lesions in the superior/middle temporal and inferior parietal gyri (Case 4). The other had left-sided lesions only in the middle frontal and superior/middle temporal gyri (Case 1). The perivascular sulcal depth lesions were noted to consist of predominantly astrocytic tau and very sparse neuronal tau, a feature that has recently been noted by others^46^ (Fig. 1).

### Clinical characteristics of cases with CTE-NC

The clinical profiles of these cases can be seen in Table 3. Four of the five cases were males, with the ages of all cases ranging from 74 to 92. In three cases the disease duration was relatively rapid at <5 years, while two other cases had longer disease durations of 8 and 15 years. The age of symptom onset ranged from 63 to 90 years of age. Most cases (except Case 4) had a clinical diagnosis consistent with a dominant dementia phenotype, with or without a movement disorder component. None of the cases had cognitive impairment or movement disorders in other family members suggestive of familial disease or had known gene mutations for neurodegenerative disease.

**Table 3:** Clinical details of cases with CTE-NC

Case number	Age at death	Disease duration (years)	Clinical diagnosis	History of TBI and contact sport participation	Cognitive impairment and/or neurobehavioural features	Disease progression	Supportive features -delayed onset (years) -motor -psychiatric	Meets traumatic encephalopathy syndrome (TES) criteria
Case 1 Female	89	4	Dementia with Lewy bodies	No	Memory impairment from age 85, increased short-term memory loss from 87. Clinical dementia rating of 1	Yes, over 4 years	Delayed onset not applicable because there was no history of repetitive head injury; no motor or psychiatric symptoms	Does not meet criteria, no known history of repetitive head injury. Clinical features explained by neuropathologically confirmed Alzheimer’s and Lewy body disease
Case 2 Male	74	8	Dementia with Lewy bodies/Alzheimer’s disease	No	Memory impairment from age 66, behaviour change; confusion and hallucinations age 71. Clinical dementia rating of 2	Yes, over 8 years	Delayed onset not applicable because no history of repetitive head injury; Akinetic rigid Parkinsonism onset age 73, Hoehn and Yahr score of 3	Does not meet criteria, no known history of repetitive head injury. Clinical features explained by neuropathologically confirmed Lewy body disease
Case 3 Male	78	15	Alzheimer’s disease/Parkinsonism	Yes, isolated TBI and former club rugby player for 20 years. Not known to have loss of consciousness	Mild cognitive impairment, anger, inappropriate behaviours diagnosed at age 63. Clinical dementia rating of 3	Cognitive decline over 15 years, movement disorder over 11 years	Yes, at least a 5-year delay of onset; Parkinsonism 11 years; depression since age 54, medicated	Meets general criteria for TES (per Table 3, this case had a subcortical infarct, hippocampal sclerosis, and LATE)
Case 4 Male	81	2	Upper limb tremor with mild cerebellar component	Yes, isolated TBI and former boxer, duration unknown. Not known to have loss of consciousness	Self-reported memory problems age 79 not found on clinical testing	No obvious progression	Yes, many years delay of onset; upper limb tremor with mild cerebellar component 2 years; self-reported depression age 80	Does not meet criteria, cognitive or neurobehavioural features not detected clinically. Short duration of movement disorder with no real progression. Duration of boxing career unknown.
Case 5 Male	92	2	Alzheimer’s disease	Yes, isolated TBI and former boxer for 5 years. Not known to have loss of consciousness	Mild cognitive impairment diagnosed at age 90. Emotional instability and mood disorder predated this diagnosis	Slight cognitive decline over 2 years; MMSE down from 26 to 25	Yes, many years delay of onset; treated for mood disorder and depression with medication age 85; and behavioural cognitive therapy age 90	Does not meet criteria for TES. Clinical features explained by intermediate Alzheimer’s disease neuropathological change

MMSE, mini-mental state examination; TBI, traumatic brain injury; TES, traumatic encephalopathy syndrome.

According to the current National Institute of Neurological Disorders and Stroke Consensus Diagnostic Criteria for traumatic encephalopathy syndrome,^16^ only Case 3 in retrospect would have met criteria for traumatic encephalopathy syndrome (see Table 3). These criteria are listed below.

1. A history of substantial exposure to repetitive head impacts, either through high contact or collision sports with a minimum 5-year history, or other non-sport-related risks for which risk thresholds have not yet been established. There were three cases that met this criterion (Cases 3–5). One played Australian rugby league at the club level for 20 years and two others had a boxing history.
2. Presence of cognitive impairment, including deficits in episodic memory and/or executive dysfunction and/or neurobehavioural features significantly different from baseline. There were four cases that met this criterion (Cases 1, 2, 3 and 5), although in three cases (Cases 1, 2 and 5) cognitive impairment can be explained by neuropathologically confirmed Alzheimer’s disease or Lewy body disease (see Point 4 below).
3. A progressive worsening of symptoms over the course of at least 1 year in the absence of continued exposure to TBI or mild repetitive neurotrauma. There were three cases that appeared to meet this criterion (Cases 3–5), although one had a comorbid diagnosis of Alzheimer’s disease (Case 5).
4. Symptoms must not be fully accounted for by other disorders, although a comorbid diagnosis of another neurodegenerative disease, neurobehavioural or substance abuse disorder can be present and does not exclude a traumatic encephalopathy syndrome diagnosis.
5. Supportive features include a delayed onset of several years after repetitive head injury ends; motor symptoms such as Parkinsonism, dysarthria and ataxia; and psychiatric features such as anxiety, apathy, depression and paranoia. Delayed symptom onset was only applicable to the three cases with a history of participation in combat and collision sports (Cases 3–5). Parkinsonism was reported in Case 3, cerebellar signs in Case 4 and mood disorder in Case 5.

The above criteria are intended to be used in a research setting to facilitate investigations into the clinical features associated with CTE-NC. Provisional levels of certainty of CTE-NC are graded as suggestive, possible, probable and definite.^16^

### Cases almost meeting criteria for CTE-NC

In addition to the five cases meeting strict consensus criteria for CTE-NC, there were eight cases that almost met criteria as they displayed astrocytic tau pathology that concentrated in an irregular pattern in the sulcal depth. However, these cases either lacked clear evidence of tau-immunopositive neurons or the pathology was not strictly perivascular (Fig. 1). These cases, therefore, met the criteria for ARTAG.^40^ All eight cases met criteria for either intermediate or high Alzheimer’s disease neuropathologic change and four also met criteria for Lewy body disease, brainstem predominant Stage IV to neocortical Stage VI. These cases were predominantly male (seven males and one female) and their ages ranged from 69 to 86 years old. Although two had disease durations shorter than 5 years, the other five cases had longer disease durations ranging from 8 to 20 years (Table 4). The age of symptom onset ranged from 55 to 76 years, which was somewhat younger than the definite CTE-NC cases.

**Table 4:** Cases almost meeting criteria for CTE-NC with coexisting pathologies

Case number	Age at death	History of TBI and contact sport participation	Gender	Disease duration (years)	Main neuropathologies
Case 1	79	No	Male	7	High AD-NC Moderate small vessel disease ARTAG LATE
Case 2	69	No	Male	5	High AD-NC ARTAG LATE
Case 3	72	No	Male	4	High AD-NC Brainstem predominant LBD ARTAG LATE
Case 4	75	No	Female	20	High AD-NC Moderate small vessel disease Hippocampal sclerosis ARTAG LATE
Case 5	80	Yes, isolated TBI and previous boxer (unknown duration)	Male	11	High AD-NC Limbic LBD ARTAG LATE
Case 6	72	Yes, isolated TBI and professional soccer for 5 years	Male	10	Corticobasal degeneration Intermediate AD-NC Hippocampal sclerosis ARTAG LATE
Case 7	76	Yes, isolated TBI	Male	8	Neocortical LBD Intermediate AD-NC ARTAG LATE
Case 8	86	Yes, isolated TBI	Male	10	High AD-NC Limbic LBD ARTAG LATE

AD-NC, Alzheimer’s disease neuropathologic change; ARTAG, ageing-related tau astrogliopathy; LATE, limbic-predominant age-related TDP-43 encephalopathy; LBD, Lewy body disease; TBI, traumatic brain injury.

All cases displayed a dementia dominant phenotype. None of the cases had cognitive impairment or movement disorder in other family members suggestive of familial disease or any known gene mutations for neurodegenerative disease, including Case 4, which had a disease onset at 55 years old. Three cases had a positive history of single TBI (Cases 6–8) and one of these also had a professional sport background as a soccer player for more than 5 years (Case 6). One case had been a boxer in his youth for an unknown time period (Case 5).

LATE was found in all cases and is commonly seen in association with Alzheimer’s disease neuropathologic change,^31^ Lewy body pathology,^47^ small vessel disease^48^ and ageing.^41^ Case 6 displayed hallmark pathological features of frontotemporal lobar degeneration with tau inclusions, specifically corticobasal degeneration. Hippocampal sclerosis was also found in Cases 4 and 6 and existed on a background of high Alzheimer’s disease neuropathologic change and corticobasal degeneration, respectively. The presence of hippocampal sclerosis has been previously documented as coexisting with CTE-NC,^18^ Alzheimer’s disease and frontotemporal lobar degeneration^31,49^ and is also a feature of LATE.^41^

### Relationship between age, TBI and neurodegenerative pathologies

In the total sample, 109 cases were identified as having a previous TBI with or without repetitive head injury from sports (17.1% of the cohort), and three (2.8% of the 109) were identified as having CTE-NC. All three of these cases were also high exposure former athletes; CTE-NC was not identified in any cases with a single TBI alone. Of the eight cases that almost met criteria for CTE-NC, four had an isolated TBI and two of these also had a history of contact sport participation (Table 4).

We used a multivariate ordinal regression model to determine how age (mean centred), gender, post-mortem delay and TBI are associated with the presence and severity of individual pathologies, specifically ß-amyloid (A Score 0–3), neurofibrillary tangles (B Score 0-3), Lewy body pathology (Lewy body Stages 0–6), cortical ARTAG (presence or absence) and LATE (presence or absence). All cases with a strong family history of disease or a known genetic mutation were removed from this analysis because these are known drivers of disease and could confound our analysis. This left a total of 507 cases for analysis (Table 5).

**Table 5:** Sporadic case types classified according to their primary neuropathological diagnosis used for regression analyses

Case type	Number of cases	% males	Mean age
Alzheimer’s disease neuropathologic change—intermediate and high	129	57.6	80 ± 12
Lewy body disease (dominant movement disorder)	99	67.4	80 ± 7
Lewy body disease (dominant dementia)	34	78.1	81 ± 9
Multiple system atrophy	21	52.4	70 ± 8
Frontotemporal lobar degeneration with TDP-43	24	60.0	71 ± 9
Motor neuron disease	41	64.6	67 ± 11
Progressive supranuclear palsy	53	56.6	77 ± 7
Corticobasal degeneration	13	53.8	73 ± 11
Pick’s disease	8	62.5	74 ± 5
Globular glial tauopathy	10	60.0	76 ± 10
Rare diseases, including frontotemporal dementia with FUS, neuronal intranuclear inclusion disease	23	50.0	83 ± 15
Cerebrovascular disease	29	55.2	88 ± 10
Healthy aged controls	21	42.9	86 ± 9
Chronic traumatic encephalopathy neuropathologic change	2	100.0	79 ± 2

Post-mortem delay was not a significant predictor of ß-amyloid pathology (A Score *P* = 0.77), neurofibrillary tangles (B Score *P* = 0.1), Lewy pathology stage (*P* = 0.19) or cortical ARTAG (*P* = 0.77). However, a greater post-mortem delay was associated with a lower likelihood of having LATE (*P* = 0.03), which may the reflect loss of TDP-43 antigenicity post-mortem. Gender was not a significant predictor of ß-amyloid pathology (A Score *P* = 0.28), neurofibrillary tangles (B Score *P* = 0.91), Lewy pathology stage (*P* = 0.15) or LATE (*P* = 0.75). However, as expected, males were more likely to have cortical ARTAG than females, with 24% of males having ARTAG compared with only 10% of females (*P* = 0.00003, see Table 6 for relative risks).

**Table 6:** Odds ratios with 95% confident intervals for gender, post-mortem delay, age and TBI on individual pathologies using multinomial regression

Pathology	OR (95% CI)	*P*-value
A score (amyloid)		
Gender	1.21 (0.85–1.71)	0.28
Post-mortem delay	0.99 (0.99–1.01)	0.77
Age	1.04 (1.03–1.06)	**0**.**0000001**
TBI	1.49 (0.98–2.29)	0.06
B score (tau)		
Gender	1.02 (0.72–1.44)	0.91
Post-mortem delay	0.99 (0.99–1)	0.1
Age	1.03 (1.01–1.05)	**0**.**00002**
TBI	1.79 (1.18–2.72)	**0**.**006**
Braak Lewy body score		
Gender	0.76 (0.52–1.10)	0.15
Post-mortem delay	0.99 (0.98–1)	0.19
Age	1.01 (0.99–1.03)	0.11
TBI	1.09 (0.69–1.71)	0.7
ARTAG		
Gender	0.29 (0.16–0.52)	**0**.**00003**
Post-mortem delay	0.99 (0.98–1.01)	0.77
Age	1.03 (1.01–1.05)	**0**.**001**
TBI	1.73 (0.97–3.07)	0.06
LATE		
Gender	1.07 (0.67–1.71)	0.75
Post-mortem delay	0.98 (0.98–0.99)	**0.03**
Age	1.09 (1.07–1.12)	**<0**.**00000001**
TBI	2.48 (1.35–4.54)	**0**.**003**

The change in relative risk represents a unit change in each independent variable. Bold values indicate statistical significance. ARTAG, ageing-related tau astrogliopathy; LATE, limbic-predominant age-related; OR, odds ratios; TDP-43,  TAR DNA binding protein 43; TBI, traumatic brain injury.

As expected, age was a significant predictor of most pathologies, showing that for every unit increase in this variable the odds of having a higher ß-amyloid stage (A score) increased by 4.3% (*P* ≤ 0.0000001), neurofibrillary tangle stage (B score) increased by 3.4% (*P* = 0.00002), and the odds of having cortical ARTAG and LATE increased by 3.3% (*P* = 0.001) and 9.4% (*P* < 0.0000001), respectively. Age was not a significant predictor of Lewy body pathology in this older adult group (*P* = 0.1, see Table 6 for relative risks).

**Figure 2:**
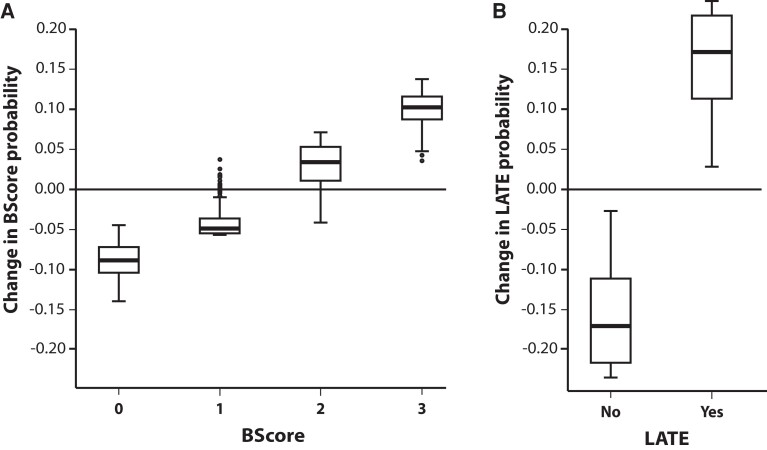
**Box plots illustrating the association between TBI and B score and TDP-43 probability**. A multivariate ordinal regression model with multivariate logit link was used to assess associations between pathological features of common neurodegenerative diseases and TBI, age (mean centred average), gender, and post-mortem delay (*N* = 507). Although TBI history was not associated with A score (amyloid), Braak Lewy body score or presence of ARTAG, both (**A**) neurofibrillary tangles (B score, *P* = 0.006) and (**B**) TDP-43 aggregation (LATE *P* = 0.003) increased significantly in these cases. The *x*- axis in (**A**) shows the four neurofibrillary tangle Stages 0–III and in (**B**) whether LATE was present or absent. The *y*-axis in both plots shows the change in predicted probability for a given outcome due to head injury. The number of data points analysed for each group for B score 0 = 117, 1 = 117, 2 = 159, 3 = 114; LATE yes = 332, no = 175.

TBI was not significantly associated with increased ß-amyloid stage (*P* = 0.06), Lewy pathology stage (*P* = 0.7) or cortical ARTAG (*P* = 0.06). However, the odds of having a higher neurofibrillary tangle (B) score increased by 79% in the TBI group (*P* = 0.006, Fig. 2A), and TBI was associated with a 148% increased likelihood of having LATE (*P* = 0.003, Fig. 2B and Table 6).

## Discussion

This is one of the largest studies examining the neuropathology of CTE published to date. In the Sydney Brain Bank, of the 636 cases screened, only five were identified as having CTE-NC, illustrating a strikingly low prevalence (0.79%). This is consistent with a recent study reporting a low frequency of CTE-NC (0.6%) in 532 individuals from a similar community-based cohort of ageing and neurodegeneration in the USA^29^ and a large-scale study reporting no CTE-NC in 310 cases from Europe.^30^ There was not a single case of CTE-NC in individuals with a documented history of a TBI alone; rather, three of the cases had TBI in addition to high exposure to repetitive head impacts in collision and combat sports and two cases had no known history of neurotrauma (TBI or repetitive head impacts in sports). All five cases had coexisting pathology, and three cases had neurodegenerative diseases diagnosed clinically and neuropathologically (Alzheimer’s disease and Lewy body dementia). There were eight cases (1.3% of the brain bank cohort) that almost met criteria for CTE-NC because they displayed astrocytic tau pathology that concentrated in an irregular pattern in the sulcal depth and in some cases could be considered to be ‘around small vessels’, but these cases either lacked clear evidence of tau-immunopositive neurons or they were not seen in a more strict and definitive perivascular arrangement (Fig. 1). Of these eight ‘CTE-like’ cases, 100% had ARTAG, LATE and Alzheimer’s disease neuropathologic change (intermediate or high). The results of our study and others suggest that CTE-NC is rare in the general population.

### Observations on the CTE-NC lesion and applying the CTE-NC consensus criteria

Thorn-shaped astrocytes consistent with ARTAG were observed in 100% of our cases with CTE-NC. However, current NINDS/NIBIB CTE consensus criteria^19^ consider subpial and subcortical astrocytic tau to be part of CTE-NC, despite being indistinguishable from ARTAG. Indeed, both are composed of 4R tau and share identical morphology.^50–52^ Increased astrogliosis and degeneration of astrocytes (beaded and broken processes) have been identified in association with CTE-NC, particularly in the white matter, which reportedly do not correlate to the amount of astrocytic tau,^53^ suggesting that another mechanism of astrocytic dysfunction might be operative. Furthermore, single nucleus RNA-seq has shown that white matter astrocytes in CTE-NC have transcript profiles consistent with neuroinflammation and dysfunctional metabolism, suggesting white matter pathology may be an important differentiating feature between ARTAG and CTE-NC.^54^ Interestingly, it was noted in the two cases with a primary pathological diagnosis of CTE-NC (Cases 3 and 4) that white matter thorn-shaped astrocytes were widespread, being present in every region examined with the exception of the cerebellum. This frequency of pathology was not observed in any of the other cases examined. The presence of astrocytic tau in all cases of CTE-NC and also those cases almost meeting criteria indicates it is likely to co-occur with the pathognomonic CTE-NC lesion, regardless of whether it is considered a supportive or separate feature. Future studies should prioritize our understanding of the differences and similarities between astrocytic tau in ARTAG and CTE-NC to better understand the relationship between these pathologies. Determining the relative significance of astrocytic versus neuronal tau in the CTE-NC lesion would also be of importance, particularly as we and others^46^ have reported the dominance of perivascular sulcal depth astrocytic tau.

Our experience of applying the NINDS/NIBIB consensus criteria and assessing various anatomical regions for pathological change supports the concept of predilection sites for CTE-NC, such as the middle frontal gyrus,^10^ but also superior/middle temporal gyri and inferior parietal gyri. However, staging CTE-NC proved more problematic. Indeed, the 2013 staging criteria^10^ have already been shown not to be consistent when used in a consensus situation in 2016^18^ or 2021,^19^ despite reported associations with age at death, presence of dementia and years of American football play^55^ in a research setting. Furthermore, the 2016 and 2021 consensus criteria acknowledge that disease staging on the background of a coexisting neurodegenerative disease,^18,19^ which is common in older individuals with CTE-NC,^20,21,56,57^ requires further investigation. Indeed, Alzheimer’s disease is one of the most frequently seen neurodegenerative diseases in cases identified as having CTE-NC,^20^ and despite the proposed differential distribution of neuronal tau in both Alzheimer’s disease and CTE-NC, the 2021 NINDS/NIBIB CTE consensus criteria^19^ does not address cases with coexisting neurofibrillary tangle pathology equal to or greater than Braak Stage IV, where tangles appear in the neocortex.^58^ This level of Alzheimer’s disease neuropathologic change is seen not only in Alzheimer’s disease but also in cases of normal ageing, making any attempt at staging difficult in aged cohorts. Previous immunohistochemical and biochemical studies have shown that neuronal tau in both CTE-NC and Alzheimer’s disease neurons consists of 3R and 4R tau that undergo common post-translational modification.^10,52,59–62^ Transcriptome analysis has also shown similar changes in protein phosphatase expression in CTE-NC and Alzheimer’s disease.^63^ However, a proteomic study has found that Alzheimer’s disease and CTE-NC share <25% homology between their insoluble proteomes, with many of the proteins identified specifically in CTE-NC also being increasingly expressed as disease stage progresses.^64^ Electron cryo-microscopy has been used to reconstruct the tau filaments in both CTE-NC and Alzheimer’s disease and has found a hydrophobic cavity exclusively in CTE-NC cases which does not associate with tau, suggesting possible different mechanisms of tau aggregation.^65^ These studies provide the necessary groundwork to identify possible key differences between neuronal tau in Alzheimer’s disease, primary age-related tauopathy and CTE-NC that may be used in a neuropathological setting to decipher the true extent of CTE-NC on the background of ageing and neurodegenerative tauopathies.

In this study, we identified a subset of cases that almost met criteria for CTE-NC except for no clear evidence of neuronal tau and/or a definitive perivascular arrangement of the lesion. These cases were mostly male and half had a known history of TBI with or without or participation in sports associated with repetitive head injury. Others have previously noted similar cases with ‘CTE-like’ pathology^27^ or with ‘features of CTE’^26^ or ‘components of CTE’^30^ and suggested they may be attributed to either restrictive sampling or may be indicative of early-stage CTE-NC that does not yet meet staging criteria. The current 2021 consensus criteria recommend that further regions be blocked bilaterally in cases of high suspicion^19^; however, despite our additional analysis of superior frontal, inferior temporal, anterior cingulate and primary motor cortex, we still did not identify any strictly perivascular neuronal lesions in these cases. Our findings, and those of others,^46^ indicate the presence of sulcal depth, perivascular thorn-shaped astrocytes is a consistent feature of CTE-NC and further research is required to better determine the significance of this pathology in the context of CTE-NC.

### Clinicopathological correlation and applying the new consensus criteria for traumatic encephalopathy syndrome

In other brain bank cohorts, CTE-NC has been previously reported in individuals with Parkinson’s disease, progressive supranuclear palsy and multiple system atrophy,^28,57^ where frequent falls are a common feature.^66,67^ However, we did not identify a single case of CTE-NC in these disease groups, which included 188 participants. As falls are reported to be the most common cause of head injury in the older population,^68^ our findings are inconsistent with speculation that falls in later life might cause CTE-NC. These findings support a previous brain bank study indicating that isolated TBIs are not associated with CTE-NC.^25^

Three of the five cases that we identified with CTE-NC had a primary neuropathological and clinical diagnosis of Alzheimer’s disease and/or Lewy body disease. Two of these cases (Cases 1 and 2) had relatively sparse foci of CTE-NC in the cortical regions examined, supporting the notion that CTE-NC was not likely the cause of dementia in individuals.^21^ In contrast, Cases 3 and 4 had more widespread CTE-NC in all cortical regions examined and extensive tau pathology in cortical and subcortical regions that was not consistent with primary age-related tauopathy due to the severe Braak stage (Stage B3).^69^ Both cases had coexisting LATE and hippocampal sclerosis, which have been previously reported in association with CTE-NC and in advanced ageing either with or without cognitive decline.^70–73^ Although the cognitive decline seen in Case 3 could be attributed to these medial temporal lobe pathologies, the presence of Parkinsonism and tremor does not fit this clinicopathological paradigm and might be more consistent with a diagnosis of traumatic encephalopathy syndrome.^16^ In contrast, Case 4 had a high level of CTE-NC but did not meet criteria for traumatic encephalopathy syndrome due to the absence of documented cognitive or neurobehavioural features and no obvious progression. Although we have a very small sample size, the data suggest that there is still considerable work to be done regarding our understanding of the clinical correlates of CTE-NC. Given that (i) the criteria for TES were published in 2021 and have not been extensively evaluated and applied in the scientific literature, (ii) we identified a very small number of cases in the brain bank that screened positively for CTE-NC and (iii) those cases had other forms of neuropathology that likely contributed in a major way to their clinical presentation, we cannot draw inferences about whether there is any association between CTE-NC and clinical findings in our cases and we cannot confirm the usefulness of the clinicopathological correlation recommendations set out in the new traumatic encephalopathy syndrome consensus criteria.^16^ It is also important to note that, while TDP-43 is considered a supportive feature of CTE-NC,^19^ it is not clear how TDP-43 associated with CTE-NC and LATE differ and how this pathology should be reported in aged cohorts where limbic TDP-43 deposition is common with or without hippocampal sclerosis.^41^

### Associations between TBI and pathology

As expected, this study found that age was a significant predictor of most pathologies. Every year of advancing age increased the odds of having a higher ß-amyloid score (A score) by 4%, neurofibrillary tangle score (B score) by 3% and the odds of having cortical ARTAG and LATE pathology by 3 and 9%, respectively. The true prevalence of ARTAG is likely to be underestimated in this study as the medial temporal lobe, a main predilection site for this pathology,^40^ was not assessed. The previous history of TBI also significantly increased the odds of a higher neurofibrillary tangle score (B score) and more than doubled the likelihood of having LATE in the Sydney Brain Bank cohort.

Some previous epidemiological studies using large population^74–78^ and military veteran^79^ cohorts, as well as meta-analyses of large numbers of observational studies,^80,81^ have suggested an association between TBI and an increased risk of Alzheimer’s-type dementia. However, post-mortem findings in this field are very mixed with many studies reporting no association between TBI and Alzheimer’s disease neuropathological change, including CERAD score,^82–84^ Thal phase^83^ and Braak stage.^82–84^ Postupna *et al*.^29^ additionally quantified the levels of paired helical filament tau, ß-amyloid 1–42, α-synuclein, phosphorylated TDP-43 as well as markers of inflammation (Iba1 and glial fibrillary acidic protein) in various brain regions of a large post-mortem cohort and found no association between TBI and these measures. However, in a subset of cases with more carefully matched control samples and more detailed analysis, higher levels of hippocampal tau were found in individuals with a history of TBI with loss of consciousness,^29^ which is more consistent with the findings from our study. Other post-mortem studies also report that TBI is associated with greater tau and ß-amyloid pathology and greater phosphorylation-independent TDP-43.^85^ Furthermore, imaging studies illustrate that tau protein accumulates following a single TBI in civilians and military service members and in former high exposure athletes in multiple brain regions^86–90^ and changes seen on tau imaging are consistent with the spatial distribution of tau pathology seen post-mortem.^89^

### Study limitations

A significant limitation of this study is that we did not systematically collect information regarding previous contact sport participation. However, Australians are traditionally active in organized sports from a young age, with an estimated 13% of the population participating in one or more of the four main contact sport codes in 2017,^91^ and more than double the amount of men playing these sports than women.^92^ It is therefore likely that we have underestimated the prevalence of mild TBI and repetitive head injury through exposure to contact sport participation in this population. That said, if we assume that substantially more people had a personal history of playing contact and collision sports, this reinforces our finding that CTE-NC is rare in the general population including in those who played sports in their youth. Additionally, the reliance on recall for the reporting of TBI and sporting history, and the non-standardized methods of data collection, particularly loss of consciousness, is a limiting factor in this study. A large meta-analysis of 15 studies and 25 134 adults reported that 12% of the adult general population has a history of TBI with loss of consciousness.^93^ We reported TBI in 17.1% of our total cohort and TBI with loss of consciousness in just under 3% of this cohort, suggesting this may have been under-reported. Additionally, our definition of TBI for this study was intentionally broad, allowing us to identify the largest sample of cases that might be at risk for CTE-NC.

Another limitation was our approach to screening. First, similar to past studies that screened large populations of cases,^26,29,30^ we examined tissue from the small number of cortical regions considered to be most valuable to detect CTE-NC.^18,19^ Given that CTE-NC can be sparse, isolated, and located nearly anywhere in the cortex, it is possible that some cases harboring this pathology were not detected. Second, both cerebral hemispheres were not screened in all cases, as recommended in both the first and second NINDS/NIBIB consensus criteria. Bilateral sampling is recommended, if possible, in order to detect patchy or isolated CTE-NC, particularly in cortical regions.^18,19^ In line with many neurodegenerative brain banks we endeavour to supply both fresh frozen and formalin-fixed specimens from cases where asymmetry of pathology is not a barrier to achieving a clear neuropathological diagnosis. Of the 636 cases analysed only 90 were sampled bilaterally to assess symmetry of CTE-NC. Of the cases with CTE-NC or ‘almost CTE-NC’ with bilateral samples, 50–60% had unilateral lesions only. It is therefore possible we may have undetected cases with CTE-NC due to the largely unilateral sampling. However, all cases identified as having unilateral pathology had less extensive and more isolated CTE-NC, which is likely to be asymptomatic.^10^

Finally, we did not determine the influence of genetic risk factors such as ApoE4 or tau haplotype on pathologies and did not record the age at injury and the time between TBI and death, which may all influence post-mortem study outcomes. Indeed, the presence of an ApoE4 allele has been shown to align with higher cortical grey matter positron emission tomography tau uptake, suggesting that individuals with this genotype may be more likely to accumulate tau pathology.^94^ Future, prospective, longitudinal studies combining clinical, genetic and post-mortem outcomes will provide the crucial information required to definitively address this area of considerable public concern.

### Conclusions and future directions

Emerging research appears to agree that single head injury is not associated with CTE-NC and many studies, including ours, indicate the frequency of this pathology in the general population is likely low.^26,29,30^ Our data support a potential role for TBI in accelerating the formation of age-related pathologies such as neurofibrillary tangles and LATE. It is unknown whether these changes represent early neurodegeneration underlying various forms of dementia, and post-mortem study outcomes in this area are still very conflicting, indicating that many factors are likely to be contributing in this complex scenario. Further studies are also required to better determine the relative contribution of neuronal and astrocytic tau to the pathognomonic lesion of CTE-NC, how to conceptualize the subset of cases that almost meet criteria for CTE-NC, and to address age-related and Alzheimer’s disease coexisting neuronal tau pathology in this context. The COllaborative Neuropathology NEtwork Characterizing ouTcomes of TBI (CONNECT-TBI) initiative^95^ has been established to address these issues, among others, and will provide an international multi-institutional effort to fill these knowledge gaps.

## Abbreviations

ARTAG =: ageing-related tau astrogliopathy
CTE =: chronic traumatic encephalopathy
CTE-NC =: chronic traumatic encephalopathy neuropathologic change
LATE =: limbic-predominant age-related TDP-43 encephalopathy
TBI =: traumatic brain injury
TDP-43 =: TAR DNA binding protein 43
